# The puzzling relationship between multi-laboratory replications and meta-analyses of the published literature

**DOI:** 10.1098/rsos.211499

**Published:** 2022-02-23

**Authors:** Molly Lewis, Maya B. Mathur, Tyler J. VanderWeele, Michael C. Frank

**Affiliations:** ^1^ Department of Psychology Carnegie Mellon University, Pittsburgh, PA, USA; ^2^ Quantitative Sciences Unit, Palo Alto, CA, USA; ^3^ Department of Psychology Stanford University, Palo Alto, CA, USA; ^4^ Harvard University, Cambridge, MA, USA

**Keywords:** meta-analysis, multi-laboratory replication, meta-science

## Abstract

What is the best way to estimate the size of important effects? Should we aggregate across disparate findings using statistical meta-analysis, or instead run large, multi-laboratory replications (MLR)? A recent paper by Kvarven, Strømland and Johannesson (Kvarven *et al.* 2020 *Nat. Hum. Behav*. **4**, 423–434. (doi:10.1038/s41562-019-0787-z)) compared effect size estimates derived from these two different methods for 15 different psychological phenomena. The authors reported that, for the same phenomenon, the meta-analytic estimate tended to be about three times larger than the MLR estimate. These results are a specific example of a broader question: What is the relationship between meta-analysis and MLR estimates? Kvarven *et al.* suggested that their results undermine the value of meta-analysis. By contrast, we argue that both meta-analysis and MLR are informative, and that the discrepancy between the two estimates that they observed is in fact still largely unexplained. Informed by re-analyses of Kvarven *et al.*’s data and by other empirical evidence, we discuss possible sources of this discrepancy and argue that understanding the relationship between estimates obtained from these two methods is an important puzzle for future meta-scientific research.

## 1. Introduction

Obtaining precise and unbiased estimates of the sizes of experimental effects is an important goal in both theory and application in psychological science. Such estimates can be used for the development and testing of quantitative models, leading to more robust theories [1]. Further, precise and unbiased estimates of intervention effects are critical for decision-making in applied contexts. Unfortunately, studies run in individual laboratories are rarely able to accumulate the sample sizes necessary to provide adequate precision [2], and furthermore individual studies may be subject to substantial publication bias. There is thus a critical need for alternative estimation methods.

Statistical meta-analysis [3,4] has long been considered a gold standard methodology for estimating effect sizes. Indeed, evidence pyramids often treat meta-analysis as one of the most credible forms of evidence, indicating the trust that is put on these quantitative summaries of the literature [5]. Yet in recent years, psychology has experienced a crisis of confidence in its prior literature, brought on by empirical reports that show low levels of replication for many prominent findings in the prior literature [6–10]. Such failures to replicate may be due in part to ‘questionable research practices’ on the part of individual researchers (e.g. *post hoc* analytic decision-making; [11]) and a bias for findings to be published only if they meet a significance threshold. Meta-analyses that include highly biased findings are suspect as sources of accurate effect estimates (or even as indicators of whether an effect is consistently non-zero; [12]).

An alternative method for estimating effects accurately is to conduct large, multi-laboratory replication (MLR) studies. Such studies provide precise estimates by enlisting many laboratories to contribute data, leading to unusually large sample sizes (by the standards of previous literature). Further, such replication attempts are typically pre-registered, reducing bias in their effect estimates by reducing analytic flexibility [13].

The presence of these two distinct routes for estimating important experimental effects naturally leads to a question: in cases of uncertainty, how much relative confidence should we place on aggregated findings using statistical meta-analysis versus large, multi-laboratory replications? In the current article, we examine a recent paper by Kvarven, Strømland and Johannesson (henceforth ‘KSJ’; [14]) that provides evidence on this issue. We conduct a series of re-analyses of their data that collectively suggest that (i) meta-analyses are in fact informative about the results of MLRs, (ii) there is a real discrepancy between the meta-analytic and MLR results (albeit less dramatic than it might seem), and (iii) publication bias probably does not fully explain this discrepancy. We end by considering alternative explanations.

## 2. Empirical comparisons of meta-analyses and multi-laboratory replications

Taking advantage of the recent increase in the prevalence of MLRs, KSJ conducted a literature review for relevant meta-analyses. They then compared effect size estimates derived from these two different methods for 15 different psychological phenomena. Naively, we might expect that if studies in a meta-analysis and the corresponding MLR assess the same psychological phenomenon, and there is no analytical or publication bias, the effect size estimates obtained via the two methods should be similar. By contrast, KSJ report that, for the same phenomenon, the meta-analytic estimate tends to be about three times larger than the MLR estimate. KSJ suggest that their results undermine the value of meta-analysis. By contrast, we argue that both meta-analysis and MLR are informative but that the relationship between them is an important puzzle for future meta-research.

To conceptualize the trade-offs between meta-analysis and MLR, it helps to consider different scenarios. In the most extreme case, in which the prior literature is stipulated to be extremely biased (perhaps due to cases of extreme analytic flexibility leading to a literature comprising only false positives), it is easy to see that meta-analysis would be worthless; MLR would be the optimal method for obtaining a precise estimate of the size of an experimental effect (for a possible example of this type, see e.g. [12]). On the other hand, if the prior literature includes some genuine positive results (even alongside some false positives), the meta-analysis will have at least some value.

Are we often in this first scenario? KSJ show that meta-analysis and MLR produce divergent estimates of effect size, but this result does not necessarily indicate that the prior literature is composed exclusively of false positives. There may be genuine and substantive reasons for differences between MLR and meta-analysis estimates. Hence, KSJ’s results do not necessarily undermine the value of meta-analysis.

**Figure 1.**
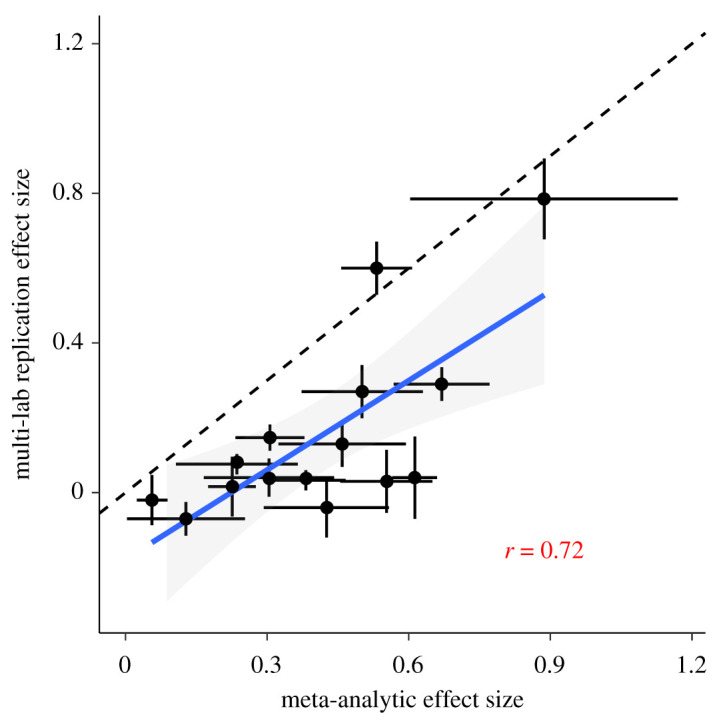
Correlation between effect size estimates from multiple-laboratory replications and random effect meta-analytic estimates (Pearson’s *r*(13) = 0.72 [0.32, 0.9], *p* = 0.003). Each point corresponds to a phenomenon (*N* = 15), and ranges indicate 95% confidence intervals. The best fitting linear model is MLR ES=−0.18+0.80 ∗ MA ES, shown here with a band corresponding to the standard error. The dashed reference line has a slope of 1.

Indeed, examining KSJ’s data indicate that there is a strong relationship between effect size estimates from the MLR and the meta-analyses: phenomena with larger meta-analytic estimates tend to also have larger estimates in the MLR (Pearson’s *r* = 0.72 [0.32, 0.90], *p* = 0.003; figure 1). Thus, although meta-analyses do show larger effects, they are not uninformative regarding the results of MLR. Hence we can infer that KSJ’s sample of findings are not generated from a world in which the prior literature is worthless—in that case, there would necessarily be no correlation between the meta-analytic estimates and the MLR estimates. Nevertheless, there appears to be a discrepancy in the size of the estimates from the two sources. What is the source of this discrepancy?

## 3. Genuine effect heterogeneity may explain some of the discrepancy

Many of the meta-analyses in KSJ’s study showed considerable effect heterogeneity. Some of this heterogeneity may be due to minor methodological differences in implementation; indeed, empirical evidence suggests that apparently minor methodological differences can substantially affect replication success.

Lewis & Frank [15] conducted a replication study of social context effects on category learning, yielding a replication estimate that was considerably smaller than that of the original study (Cohen’s *d* = 0.17 versus *d* = 1.49), as in many of KSJ’s comparisons. However, in a series of four subsequent replications, Lewis & Frank [15] identified and eliminated a handful of minor methodological differences between their original replication design and the original study (such as the variability in category exemplars). The final replication, with the fewest known methodological differences from the original study, estimated a fairly large effect size of *d* = 0.71, which was more than four times that of the first replication study (*d* = 0.17), although the estimate remained smaller than that of the original study. The estimated heterogeneity across the five replication studies, *τ*^2^ (0.04), was within the range found in the sample of meta-analyses reported by KSJ (0–0.54, with a mean of 0.11). Notably, we observed this effect size variability in a case where the experiment was motivated by a formal model [16], and where the methodological modifications were theory-irrelevant (e.g. online versus in laboratory; exact stimuli used). The fact that we find this variability, despite the tight link between theory and experiment [1], is particularly suggestive that small methodological decisions about the implementation of individual experiments can substantially influence the meta-analytic effect size. It therefore seems plausible that similar methodological differences could be fairly common in MAs.

Importantly, when effects are heterogeneous, a comparison of the meta-analytic mean to the MLR mean—KSJ’s primary comparison—does not, on its own, adequately characterize the evidence for a true difference between the two [17]. Comparing only the means of potentially heterogeneous effect distributions cancreate a false impression of conflict when in fact little conflict exists because the MLR mean could fall within the reasonable distribution of effects in the meta-analysis [17,18].

To test this idea, we examined where each MLR mean would fall in the distribution of effects in the corresponding meta-analysis. A naive approach to this question would simply look at the variability of effects. However, this approach would overstate variability in true effects because it incorporates both variability in the true effects measured by different studies *and* variability due to noise in individual studies.

To address this issue, we used statistical methods that estimate the distribution of population effects in a meta-analysis while accounting for statistical error in the point estimates [17,19]. To do so, one first fits a standard meta-analysis, then uses the resulting estimates to appropriately ‘shrink’ studies’ point estimates toward the mean (to account for statistical error), and then finally uses the empirical distribution of these shrunken estimates as an estimate of the distribution of population effects [17,19,20]. (This shrinkage is necessary because the distribution of the point estimates themselves has variability due to both statistical error and heterogeneity, so cannot be directly used to characterize heterogeneity in the population effects.)

**Figure 2.**
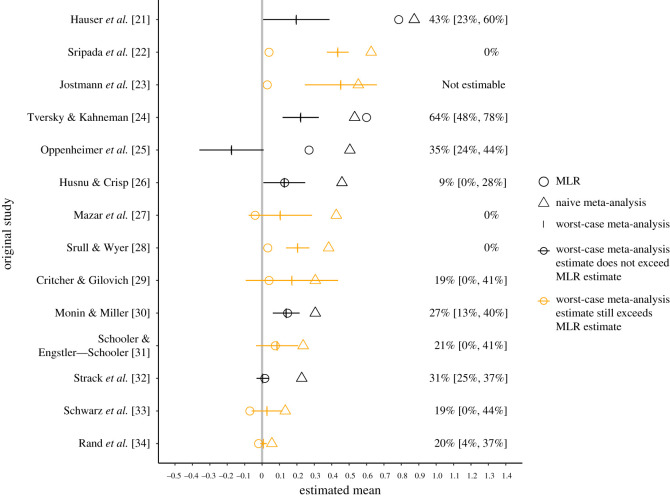
The text values on the right represent estimated percentages and corresponding 95% CIs of true population effects in the naive meta-analysis that are as small as, or smaller than, the MLR estimate. CIs are omitted when they were not estimable via bias-corrected and accelerated bootstrapping [19]. The left side of the figure shows estimates from sensitivity analyses representing worst-case publication bias (vertical tick marks) versus naive meta-analysis estimates (triangles) and multi-laboratory replication estimates (MLR; circles). For orange-coloured meta-analyses, the worst-case estimate exceeds the MLR estimate, indicating that no amount of publication bias that results could entirely explain the discrepancy between the naive estimate and the MLR estimate.

We thus estimated that, across the meta-analyses that KSJ analysed, a median of 20% of the population effects in the meta-analysed studies were at least as small as the corresponding MLR mean estimate (see right side of figure 2 for estimated values for each MA). For comparison, if 50% of the population effects were at least as small as the corresponding MLR mean, then the MLR mean would be at the meta-analytic population median. Thus, although *average* effect sizes were typically larger in meta-analyses versus MLRs, often a sizeable minority of the effects in the meta-analyses were smaller than the corresponding MLR estimate. This finding indicates a smaller discrepancy than is apparent when comparing only the means.

This analysis reveals that, when we holistically consider the distribution of effects rather than only its mean, there is a non-negligible amount of overlap between the distribution of possible effects in a meta-analysis and the MLR results. But although the discrepancy between meta-analysis and MLR results is perhaps smaller than KSJ suggest, a discrepancy does still exist. Effect heterogeneity alone does not fully account for the differences between the two approaches. The remainder of our discussion focuses on explaining these differences.

## 4. Publication bias cannot entirely explain the discrepancy

KSJ speculate that the MLR/meta-analysis discrepancy is probably due to ‘questionable research practices’ [11] such as *post hoc* analytic decision-making or publication bias. Both of these practices act as filters that select for statistically significant findings, leading to an inflation of effect size. Could these mechanisms be fully responsible?

One way to address this question is to estimate the meta-analytic effect size using a correction for publication bias. KSJ made this estimate using several different statistical methods; each of these still yielded estimates that were considerably larger than the MLR estimates. KSJ therefore concluded that the statistical methods are themselves flawed and ‘ineffective in fully adjusting inflated effect sizes for publication bias’.

In our view, this analysis presupposes its conclusions. The conclusion that the methods are flawed because they do not eliminate the discrepancy itself requires assuming that the discrepancy is due to publication bias rather than other possibilities. In fact, there are several different possibilities. That principled statistical adjustments [35] do not eliminate the systematic discrepancy between the meta-analysis and MLR estimates could reflect either: (i) that statistically adjusted meta-analysis estimates were indeed badly biased due to serious violations of the methods’ assumptions, or alternatively (ii) that the statistically adjusted meta-analysis estimates were not, in fact, badly biased, because there are fundamental substantive reasons, not merely publication bias, for effect sizes to genuinely differ between meta-analyses and MLRs.

To help adjudicate between these two possibilities, we conducted a re-analysis that uses a different approach for assessing publication bias, called ‘sensitivity analysis’ [36]. In contrast to the methods used by KSJ, the sensitivity analysis method corrects the estimate not by attempting to estimate the actual severity of publication bias present in the meta-analysis, but rather by considering only a hypothetical worst-case form of publication bias. In a nutshell, this sensitivity analysis ignores *all* statistically significant results in the expected direction [36]. Heuristically, the logic of this worst-case analysis is that all of these estimates might be simply due to publication bias. By contrast, sensitivity analysis considers *only* effects that are non-significant or in the unexpected direction, which could not have been published due to publication bias. Thus, sensitivity analysis provides a highly conservative estimate and obviates many (though not all) of the assumptions of standard methods that might in principle have caused them to adjust inadequately [36].

For our sensitivity analysis, we assume a model of publication bias in which statistically significant positive studies (affirmative studies) are more likely to be published than non-significant and/or negative studies (non-affirmative studies), and there is no further selection based on the size of the point estimate or on characteristics associated with the point estimate (such as the *p*-value treated as continuous, rather than dichotomized at *α* = 0.05). This model of publication bias is identical to that assumed by the three-parameter selection model^1^ used by KSJ [35], and it conforms well to empirical evidence regarding how publication bias operates in practice [11,37,38].

However, unlike the three-parameter selection models used in KSJ, the present methods do not require a large number of meta-analysed studies to perform well [39,40] and do not make any distributional assumptions (e.g. that the population effects are normal prior to the introduction of publication bias). ‘Publication bias’ in this context could reflect the aggregation of multiple sources of bias, including, for example, investigators’ selective reporting of experiments or preparation of papers for submission as well as journals’ selective acceptance of papers.

To provide some more intuition for how the sensitivity analysis methods work, if the degree of publication bias were known, a bias-corrected meta-analytic estimate could hypothetically be obtained by up-weighting the contribution of each non-affirmative study in the meta-analysis by the same ratio by which the publication process favours affirmative studies. For example, suppose it were known that affirmative results were five times more likely to be published than non-affirmative studies and that, given a study’s non-affirmative or affirmative status, the publication process did not select further based on the size of the point estimate. Then the point estimates of the published non-affirmative studies (i.e. those included in the meta-analysis) would be essentially a random sample of those from the larger, underlying population of non-affirmative studies, of which most were not published. A bias-corrected meta-analytic estimate could therefore be obtained by up-weighting the contribution of each non-affirmative study in the meta-analysis by fivefold to counteract the publication process’ fivefold favouring of affirmative studies.

Since the degree of publication bias is not exactly known in practice, the sensitivity analyses can also estimate the meta-analytic mean under a hypothetical ‘worst-case’ publication bias scenario [36], in which affirmative studies are *infinitely* more likely to be published than non-affirmative studies. That is, worst-case publication bias would effectively favour affirmative studies by an infinite ratio, so a worst-case estimate can be obtained by meta-analysing *only* the non-affirmative studies that are included in the meta-analyses and simply discarding the affirmative studies. Intuitively, this method works because such an analysis is effectively equivalent to up-weighting each non-affirmative study by a factor of infinity. Such a worst-case analysis does not require actual estimation of the ratio by which the publication process favours affirmative studies.

We conducted this sensitivity analysis for the KSJ data by analysing 13 of the 15 meta-analyses for which the meta-analytic mean estimate was larger than the MLR estimate and for which these analyses were statistically feasible (meta-analyses must contain at least one non-affirmative study). Across the meta-analyses, the mean naive and worst-case estimates were *d* = 0.39 and *d* = 0.17, respectively. Thus, the worst-case estimates were on average 32% as large as their corresponding naive estimates, with a mean absolute difference of *d* = 0.25. Nevertheless, for the majority of such meta-analyses (62%), even worst-case publication bias of this nature could not attenuate the meta-analytic estimate to match that of the MLR. That is, even selecting only the non-significant findings for the meta-analysis led to a meta-analytic estimate larger than the MLR estimate! Also, for all but one of these meta-analyses (i.e. 92%), worst-case publication bias could not attenuate the meta-analytic estimate all the way to the null (figure 2).

It therefore appears somewhat implausible that publication bias, no matter how severe, could entirely explain the discrepancy between meta-analytic and MLR effect size estimates. This suggests that KSJ’s failure to eliminate the discrepancy in effect sizes derived from the two methods was not due entirely to limitations of publication bias correcting statistical methods; rather, there are additional causes of the discrepancy.

## 5. Possible explanations beyond publication bias for the remaining discrepancy

What, then, are the other possible causes of the discrepancy? One possibility is that the phenomena being studied may be sensitive to the details of the experimental materials and methods, and especially to how these interact with the specific populations being assessed. This kind of method- and context-sensitivity has frequently been invoked as a *post hoc* explanation for direct replication failures [41], but direct evidence has been limited. Nevertheless, we are sympathetic to this explanation because a series of pre-registered replications that we and our collaborators have carried out attest to the importance of small methodological factors in replicating effects [15,42,43]. For example, in [15], we found that a series of minor changes in stimulus materials (e.g. variability of exemplars) led to differences in the strength of a categorization effect.

Further, subtle methodological choices made by individual studies within a meta-analysis may interact with the population of participants in those experiments. (A version of this point is made by Yarkoni [44] as well.) Investigators who are committed to understanding a particular phenomenon may take pains to tailor their stimuli to that particular context, via pilot testing or application of their intuitions about the specific participant population. Probably, this is one reason that the individual studies in a meta-analysis typically vary considerably in their methods and stimuli. By contrast, MLRs typically standardize their materials across all populations and contexts being studied in order to establish a single method for all participating laboratories. This difference—variable materials versus standardized materials—could account for some of the discrepancy in effect sizes.

For example, one of the phenomena included in KSJ’s paper is an effect whereby imagining interaction with an outgroup member leads participants to be more likely to express an intention to engage with an outgroup member in real life. In the original paper [45], the participants were British non-Muslim undergraduates, and the ‘outgroup’ was British Muslims. In the corresponding MLR, 34 out of the 35 replication sites used the same outgroup, ‘Muslim', despite the fact that the sites spanned nine different countries with probably varying degrees of prejudice toward Muslims. By contrast, in the meta-analysis, individual studies used a wide range of outgroups, adapted to the local social context of each study. Furthermore, even if laboratories were to likewise alter the stimuli according to context, individual studies may be more likely than MLRs to select samples and exclusion criteria to maximize effect sizes.

In social psychology especially, effects could well be more context sensitive relative to other psychological domains [41,46]. Notably, the majority of phenomena in KSJ concern effects that appear social or contextually dependent (e.g. interactions between political belief and moral decision-making, humor responses, imagined intergroup contact, expression of prejudice). However, to carry empirical weight, speculations about context-sensitivity must be tested directly in future meta-scientific work.

A final hypothesis about the discrepancy between meta-analysis and MLR may be that in individual studies, as compared with MLRs, investigators may make greater (or more effective) efforts to ensure intervention fidelity, thereby increasing effect sizes. Such differences in fidelity would not constitute investigator bias; instead, under such circumstances, the interventions themselves would effectively be different (e.g. because participants received greater encouragement to engage). Such differences could be due to experimenter expertise, though meta-scientific attempts to find effects of expertise on replication success have been unsuccessful [6]. More plausibly in our mind, differences could be due to feeling of ‘having more at stake’ by original investigators relative to the myriad teams participating in a MLR effort, who may assume that a protocol being distributed to them should ‘just work’. (We write this characterization as participants in a variety of MLR efforts.)

## 6. Conclusion

Building good scientific theories relies on having precise estimates of effect sizes, but the best way to obtain these estimates is not obvious. Both meta-analysis and MLRs provide methods for estimating the effect size of important phenomena by aggregating evidence across multiple studies. KSJ present the first systematic comparison of these two methods and show that effect sizes derived from meta-analyses are puzzlingly larger than those derived from MLRs. We demonstrate that meta-analytic effect sizes are related to MLR estimates, but there is still a remaining discrepancy between the two methods. Further, our analyses suggest that effect size heterogeneity and publication bias may contribute to—but are unlikely to account fully for—this discrepancy. Speculative possibilities for the remaining discrepancy include that MLRs obtain smaller effect sizes because of standardization of methods across laboratories (perhaps especially for context-sensitive phenomena) and because of the potential for differential effort to ensure intervention fidelity comparing MLRs and original literature. Understanding the source of the discrepancy between effect sizes estimated from meta-analyses and those from MLRs is an important, complex question for future meta-scientific research.

## Footnotes

1 Three-parameter selection models assume that studies with ‘significant’ positive estimates are more likely to be published than studies with ‘non-significant’ or negative estimates. These models use maximum likelihood to model this form of publication bias, jointly estimating the meta-analysis mean, heterogeneity, and the severity of publication bias operationalized as the ratio of publication probabilities for ‘significant’ positive versus ‘non-significant’ or negative results.

## Data accessibility

The data are provided in electronic supplementary material [47]. Data and relevant code for this research work are stored in GitHub: https://github.com/mllewis/kvarven_reanalysis and have been archived within the Zenodo repository: https://zenodo.org/record/5822514#.YdXOvH3MI3E.
